# First detection of *Colpodella* spp. in *Rhipicephalus annulatus* and molecular characterization of piroplasmids in southern Egypt

**DOI:** 10.3389/fvets.2025.1617204

**Published:** 2025-06-18

**Authors:** Ahmed M. Soliman, Hassan Y. A. H. Mahmoud, Moaz M. Amer, Samah Mohamed, Tatsuro Hifumi, Kyoko Tsukiyama-Kohara, Tetsuya Tanaka

**Affiliations:** ^1^Laboratory of Infectious Diseases, Joint Faculty of Veterinary Medicine, Kagoshima University, Kagoshima, Japan; ^2^Transboundary Animal Diseases Center, Joint Faculty of Veterinary Medicine, Kagoshima University, Kagoshima, Japan; ^3^Biotechnology Department, Animal Health Research Institute, Agricultural Research Center, Giza, Egypt; ^4^Division of Infectious Diseases, Animal Medicine Department, Faculty of Veterinary Medicine, South Valley University, Qena, Egypt; ^5^Faculty of Medicine, Mansoura University, Mansoura, Egypt; ^6^Laboratory of Veterinary Histopathology, Joint Faculty of Veterinary Medicine, Kagoshima University, Kagoshima, Japan; ^7^Laboratory of Animal Microbiology, Graduate School of Agricultural Science, Tohoku University, Sendai, Japan

**Keywords:** *Colpodella* spp., *Babesia bovis*, *Theileria* spp., *Hyalomma dromedarii*, *Hyalomma marginatum*, *Rhipicephalus annulatus*, cattle, Egypt

## Abstract

Piroplasmosis is a tick-borne disease that can affect livestock, particularly cattle. Its causative pathogens are intracellular apicomplexan parasites belonging to the order Piroplasmida. We recently identified one such emergent pathogen (*Colpodella* spp.) in ticks infesting camel in Egypt. Accordingly, we aimed to ascertain the presence of hemoprotozoan parasites in ticks infesting cattle. We removed ticks from household cattle during veterinary examinations, and submitted them for morphological examination and PCR analyses for species identification. Ticks and hemoprotozoan species obtained from tick samples were also evaluated using BLAST analysis, followed by confirmatory phylogenetic analyses. The collected ticks were identified as belonging to three species: *Hyalomma dromedarii, Hyalomma marginatum*, and *Rhipicephalus annulatus*. Phylogenetic analysis based on the 16S rRNA gene revealed that these ticks were clustered with those of the relevant species previously documented in Egypt. Molecular analysis targeting the 18S rRNA gene revealed *Colpodella* spp., the second such report in Egypt and the first in *R. annulatus* ticks infesting cattle. The *Colpodella* minimum infection rate (MIR) was 2.3% (per sample of pooled ticks from a single bovine host). Furthermore, *Babesia bovis, Theileria. annulata*, and *Theileria orientalis* were detected with MIRs of 3.5%, 4.7%, and 0.39%, respectively. In the phylogenetic analysis, each detected pathogen clustered with its corresponding species. Specifically, the *Colpodella* spp. were grouped with *Colpodella* spp. previously detected in *Rhipicephalus microplus, Rhipicephalus haemaphysaloides* ticks, and humans in China (accession numbers MH208620, MH208621, and GQ411073), and *H. dromedarii* ticks infesting camel in southern Egypt (accession numbers LC775361 and LC775361). We confirmed the detection of *B. bovis* and *T. annulata* through PCR assays with specific primers targeting the *spherical body protein-4* gene and the *major merozoite surface antigen* gene, respectively. The detection of *Colpodella spp*. in ticks infesting cattle highlights the need for ongoing surveillance of this parasites. Both cattle and camels may serve as sentinel species, emphasizing the importance of monitoring these livestock for emerging parasites.

## 1 Introduction

Piroplasmosis is a disease caused by intracellular apicomplexan parasites in the order Piroplasmida, mainly *Theileria* or *Babesia* spp. (1). The primary vectors are ixodid ticks, which are known to pose a significant threat to human and animal health as hematophagous ectoparasites that transmit pathogens when they feed on a host's blood (2). Beyond the threat to health, ticks and the protozoan pathogens they transmit are a major cause of economic loss in the agricultural sector, as tick-infested livestock are exposed to the pathogens that cause piroplasmosis. Infected livestock may show classic tick-borne disease symptoms such as weight loss and progressive anemia, and adverse effects on the udder, skin, and hide, and may die or have to be slaughtered (3, 4).

Piroplasmosis has been detected in various provinces of Egypt, suggesting that large parts of the country are prone to outbreaks of tick-borne diseases (5–9). Recent reports have highlighted newly emergent tick-borne protozoa in Egypt; specifically, *Babesia naoakii* was detected in camels (10), and a *Colpodella* spp. was detected in *Hyalomma dromedarii* ticks collected from camels (11). These findings demonstrate the importance of monitoring the distribution of tick species and tick-borne pathogens in livestock across Egypt. Although camels are commonly used in Egyptian agriculture, cattle are the predominant livestock species across the whole country; thus, there is a need to extend monitoring for the pathogens recently identified in ticks infesting camel to cattle.

Of the newly detected protozoan parasites in Egypt mentioned above, *Colpodella* spp. was the most recently reported, and is particularly worthy of attention as the potential epidemiological implications for human and animal health are not yet well understood. *Colpodella* spp. are a group of small predatory flagellates (12). They are predominantly free-living microorganisms that typically feed on algae or protozoa, and have only occasionally been found in vertebrates and arthropod vectors (13, 14). The first human case of *Colpodella* spp. infection was reported in China in 2012. In that case, cell morphology exams (Giemsa staining) revealed multiple, ring-like forms infecting erythrocytes, and the detection of a *Colpodella* spp. was formally confirmed through molecular analysis (15). Additionally, a *Colpodella* spp. has been identified in *Rhipicephalus microplus* ticks infesting cattle in Mozambique, Africa (16). In 2018, *Colpodella* spp. was found in ticks, and in a human patient exhibiting neurological symptoms, at the same location as that of the first human case in China (Qinghai Province). Notably, *Colpodella tetrahymenae* and *Colpodella* spp. were obtained in that report, and four sequences were identified (accession numbers MH012044-MH012047) (17). Recently, in China, *Colpodella* spp. have been reported in horses (18), and in Amur tigers (*Panthera tigris altaica*) and the ticks attached to them (19). Our previous report on *Colpodella* spp. was made in camels in the Luxor and Aswan regions of Upper Egypt. The spread of this previously little-reported pathogen from East Asia to North Africa is a cause for concern, especially considering the severity of the symptoms documented in the human case.

In Egypt, the prevailing climatic conditions provide a highly favorable habitat for numerous tick species, and the control measures against the potential threat to public health and the livestock-based economy remain insufficient (20). Thus, there is a pressing need to correct the paucity of epidemiological data on tick species and the protozoan parasites they transmit, especially in cattle populations, which represent the mainstay of Egyptian agriculture. Thus, the primary goal of this research was to identify tick species infesting cattle and any protozoa present in these ticks, in three governorates in Upper Egypt (including an area where *Colpodella* spp. had previously been detected) using PCR-sequencing assays, to provide crucial information for developing effective strategies to combat tick-borne diseases.

## 2 Materials and methods

### 2.1 Study area and design

**Figure 1:**
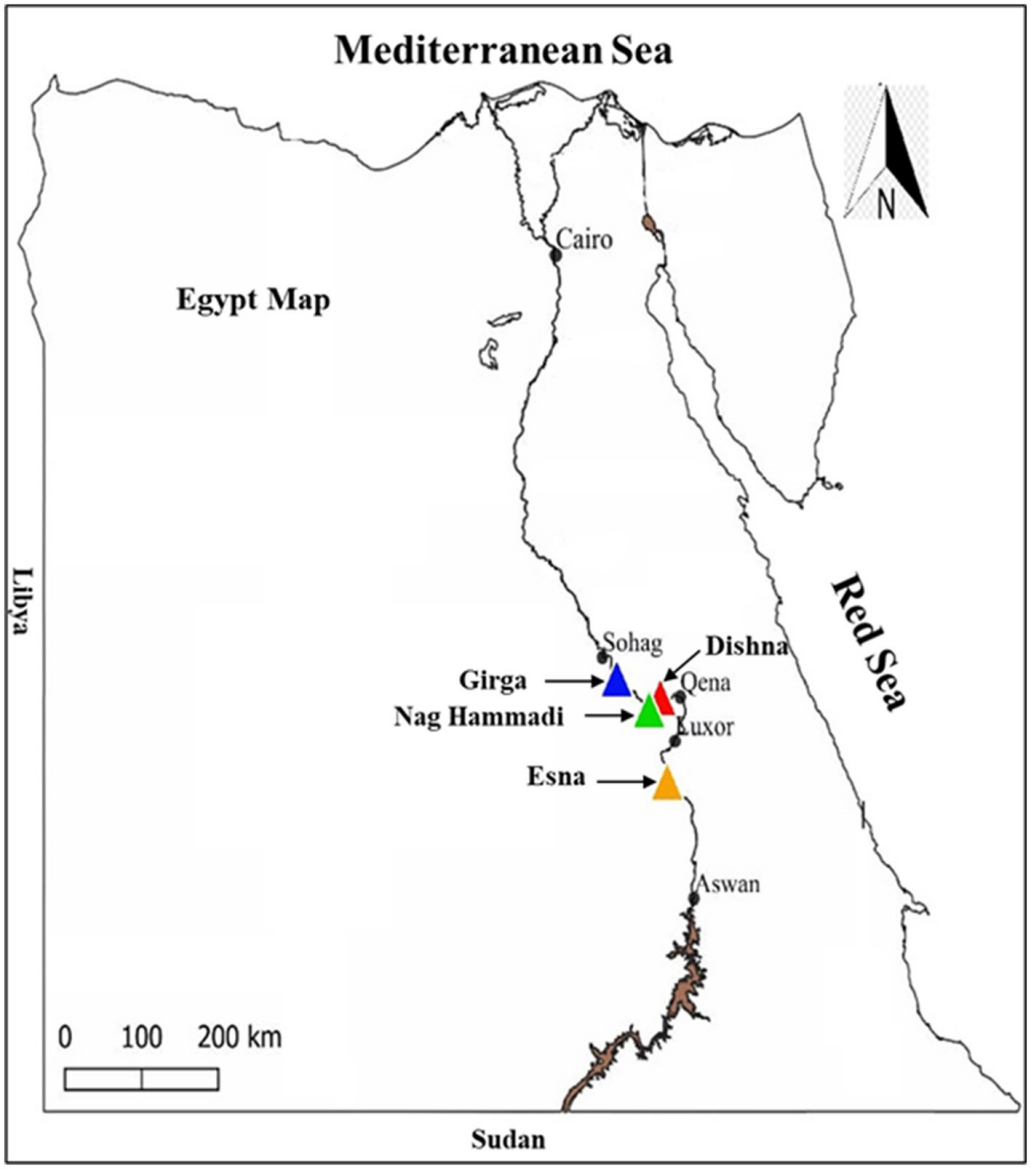
Locations targeted for tick collection in Egypt.

This cross-sectional study was conducted in four districts across southern Egypt: Deshna (26°7′18.595"N, 32°28′17.511"E) and Naja' Hammadi (26°2′55.6"N, 32°14′25.12"E) in the Qena governorate, Girga (26°20′13.96"N, 31°53′34.6"E) in the Sohag governorate, and Esna in the Luxor governorate (25°17′24.3"N, 32°33′23"E). These areas were selected based on their high livestock densities and documented history of tick infestations and tick-borne diseases, making them epidemiologically relevant for studying tick-pathogen dynamics. The hot and arid climate of southern Egypt, characterized by minimal annual rainfall and elevated temperatures, provides favorable conditions for tick survival and pathogen transmission. The geographical positions of the study areas within southern Egypt are shown in Figure 1.

The current study was conducted to investigate the presence and molecular diversity of tick species and associated piroplasmids in southern Egypt. The aim was to provide baseline data on tick infestation patterns and tick-borne protozoa with a focus on identifying emerging or underreported tick-borne pathogens. Collected ticks were morphologically identified and pooled according to species, developmental stage, and geographic origin for molecular analysis targeting piroplasmids and related protozoan parasites. All laboratory procedures, including DNA extraction, PCR amplification, and sequencing, were performed using standardized protocols to ensure reproducibility and accuracy.

### 2.2 Tick collection and tick morphological identification

A total of 110 cattle were selected for tick collection during routine veterinary inspections conducted between January and July 2019. The sample size was determined based on accessibility and logistical constraints, with the aim of generating preliminary baseline data on tick species composition and associated pathogens in the study area. We attempted to collect a uniform number (2, 3) of ticks from each bovine host. All ticks were removed manually from the medial aspect of the thighs, udder, dewlap, or axilla, taking care to minimize any discomfort to the animal. Sampling was not randomized or stratified due to logistical constraints and the observational nature of the study; however, efforts were made to include animals from multiple locations within each location to attain variability in tick populations. The owner of each cattle provided informed consent for the collection of ticks from their cattle, and the use of the samples in this study. The number of collected ticks was recorded for each cattle at each location. After collection, the ticks were preserved in tubes containing 70% ethyl alcohol. Each tick was subjected to a cleaning process before species identification by microscopy. The procedure involved placing the tick in a fine mesh and subjecting it to a gentle stream of tap water to eliminate surface dirt and debris. Subsequently, the ticks were immersed in 70% ethanol for 2 min and then rinsed twice in sterile Milli-Q water. Ticks were cleaned thoroughly to enhance the visibility of their taxonomic characteristics. Tick species were identified using standard identification keys (21–24).

### 2.3 Tick processing and DNA isolation

Ticks underwent an initial cleansing process with 70% ethanol and were washed with sterile Milli-Q water before air-drying. Each tick was then placed in a 2-ml tube also containing a stainless-steel bead for subsequent crushing, following overnight freezing at −80°C. The crushing procedure utilized an Automill crusher (Tokken. Inc, Japan) for three cycles, each lasting 30 s at 2,000 rpm. Following crushing, 200 μl of 1 M Tris-HCl (pH = 7.5) was added to each sample tube, and the tube was well shaken for 15 min, to ensure thorough mixing of the contents. After removing the stainless-steel beads, the tubes were centrifuged at 14,000 g for 5 min at 4°C. A 200-μl amount of tick homogenate was then carefully collected for DNA isolation.

The tick samples collected from each animal were pooled according to species, life cycle stage, and sampling area before DNA extraction. One hundred seventy-seven pools were generated based on sample size (1– 3 ticks/pool). The DNA isolation process employed a fully automated nucleic acid extractor, (magLEAD 6gc, Precision System Science Co., Ltd., Chiba, Japan), employing MagDEA^®^ Dx SV (Precision System Science Co., Ltd., Chiba, Japan), and was conducted following the manufacturer's instructions. This method was chosen over manual extraction due to its higher efficiency, reduced contamination risk, and improved reproducibility, ensuring standardized DNA yields. The DNA concentration was determined using a Nanodrop^TM^ 2,000 spectrophotometer (Thermo Fisher Scientific, Waltham, MA, USA). The DNA samples were stored at −30°C for subsequent analysis.

### 2.4 Molecular characterization of ticks

Samples were then subjected to a conventional PCR assay to confirm the existence of tick genomic DNA and as assure the absence of PCR inhibitors. In this assay, we targeted a specific partial fragment of the 16S rRNA gene unique to tick species (25) for amplification. The PCR mixture was prepared at a total volume of 10 μl, and comprised 5 μl of 2 × Gflex PCR buffer (containing Mg2^+^, dNTP plus; Takara Bio Inc., Kusatsu, Shiga, Japan), two 0.5 μl primer solutions (one each for the forward and reverse primers) with a concentration of 10 μm, 0.5 μl of Tks Gflex^TM^ DNA polymerase (1.25 U/μl; Takara Bio Inc.), 3.2 μl of Milli-Q water, and 0.5 μl of the extracted DNA template, ranging in concentration from 10 to 30 ng/μl. The sequences and annealing temperatures of the used primers are stated in Table 1. Nuclease-free water was utilized as a negative control and template for contamination monitoring. A confirmed NA sample for *Haemaphysalis longicornis* that had been stored in our laboratory and subject to confirmatory sequencing was utilized as the positive control, and exhibited a DNA concentration of 10 ng/μl.

**Table 1:** Primer sequences used in this study.

**Pathogen (target gene)**	**Assay**	**Primer sequences (5** ^**′**^ **-3** ^**′**^ **)**		**Ta (°C)**	**Size (bP)**	**Ref**.
		**Forward**	**Reverse**			
Tick (16S rRNA)	PCR	CTGCTCAATGATTTTTTAAATTGCTGTGG	CCGGTCTGAACTCAGATCAAGTA	56	455	(25)
Piroplasmid (18S rRNA)	nPCR	GGTGAAACTGCGAATGGCTC	AAGTGATAAGGTTCACAAAACTT	55		(26)
		TGGCTCATTACAACAGTTATA	CGGTCCGAATAATTCACC	55	1,550	
*B. bovis* (^*^sbp-4)	nPCR	AGTTGTTGGAGGAGGCTAAT	TCCTTCTCGGCGTCCTTTTC	55	503	(27)
		GAAATCCCTGTTCCAGAG	TCGTTGATAACACTGCAA	55		
*B. bigemina* (^*^RAP1a)	nPCR	GAGTCTGCCAAATCCTTAC	TCCTCTACAGCTGCTTCG	55	412	(27)
		AGCTTGCTTTCACAACTCGCC	TTGGTGCTTTGACCGACGACAT	55		
*T. annulata* (^*^Tams 1)	PCR	GTAACCTTTAAAAACGT	GTTACGAACATGGGTTT	55	721	(28)

^*^mt-rrs, mitochondrial 16 S rRNA; ^*^SBP-4, spherical body protein 4; ^*^RAP1a, rhoptry-associated protein1; ^*^Tams 1, T. annulata merozoite surface antigen; Ta, annealing temperature.

### 2.5 Molecular detection of piroplasms

A nested PCR assay was used to detect the 1,550-bp fragment of the 18S rRNA gene specific to piroplasms (26). Moreover, we employed nested PCR assays to pinpoint gene fragments specific to pathogens: the 503-bp fragment of the spherical body protein-4 (*SBP-4*) gene for *B. bovis* (27), and the 412-bp fragment of the rhoptry-associated protein 1 (*RAP1a*) gene for *Babesia bigemina* (27). Additionally, a conventional PCR assay was conducted to detect the 721-bp fragment of the major merozoite surface antigen (*Tams1*) gene, which is specific to *T. annulata* (28). The PCR mixture was prepared at a total volume of 10 μl. It included 5 μl of 2 × Gflex PCR buffer (Mg^2+^, dNTP plus) (Takara Bio Inc.), two 0.5 μl primer solutions (one each for the forward and reverse primers) at a concentration of 10 μm, 0.5 μl of Tks Gflex^TM^ DNA polymerase (1.25 U/μl) (Takara Bio Inc.), 3.2 μl of Milli-Q water, and 0.5 μl of the extracted DNA template with a concentration ranging from 10 to 30 ng/μl. In the nested PCR assays, 0.5 μl of the first PCR product (diluted 25-fold) was used as the template in the second PCR reaction. The sequences and annealing temperatures of the used primers are provided in Table 1. All PCR assays included strict measures for contamination monitoring, and employed nuclease-free water as the negative control. Additionally, confirmed DNA samples for each pathogen (*Piroplasma* spp., *B. bovis, B. bigemina*, and *T. annulata*) that had been obtained from Egyptian cattle blood and stored in our laboratory were used as positive controls. These positive control samples had undergone confirmatory sequencing, and they demonstrated DNA concentrations ranging from 10 to 15 ng/μl.

The PCR products obtained from the assays above were subjected to electrophoresis on a 1.5% agarose gel in 1 × Tris-acetate-EDTA (TAE) buffer. Gel electrophoresis was performed using a Mupid electrophoresis device (Mupid Co., Ltd., Tokyo, Japan). After electrophoresis, the DNA bands were visualized using a gel documentation system with a UV device (WUV-M20; ATTO Co., Ltd., Tokyo, Japan). Bands were visualized after staining the gel with ethidium bromide at a 5 μg/ml concentration in 1 × TAE buffer.

### 2.6 Sequencing and phylogenetic analysis

The positive amplicons obtained from each gene detection were subjected to purification from the agarose gel, employing the NucleoSpin Gel and PCR Clean-up kit (Macherey-Nagel, Leicestershire, Duren, Germany), in accordance with the manufacturer's instructions. Subsequently, the purified DNA samples were sent to a commercial laboratory (Eurofins NSC Japan KK, Kanagawa, Japan) for sequence analysis using a 3730 × 1 DNA Analyzer (Thermo Fisher Scientific, Waltham, Massachusetts, USA). The bi-directional sequencing results were first evaluated using SnapGene Viewer software (GSL Biotech, LLC., Boston, USA) (https://www.snapgene.com/). The forward and reverse sequences were aligned and combined using MEGA 11 software to produce complete sequences for subsequent analysis.

To validate the identification of the tick-borne protozoa, the obtained sequences were compared with reference sequences in the GenBank database utilizing the BLASTn tool. Obtained sequences were aligned with reference sequences from the GenBank database, using the ClustalW algorithm in MEGA 11 software. After alignment, the sequences were trimmed, and a model test was conducted in MEGA 11 to identify the most suitable evolutionary model for the data. Subsequently, phylogenetic trees were constructed using the Maximum Likelihood method, with the Tamura 3-parameter model applied for identifying tick species, *B. bovis SBP4* gene, and *T. annulata Tams-1* gene. For the detection of *Protozoal* spp. 18S rRNA genes, the Tamura-Nei model was used as the evolutionary model. Tree reliability was evaluated by bootstrap analysis with 1,000 replicates (29).

### 2.7 Statistical analysis

The minimum infection rate (MIR) was calculated as the number of positive pools divided by the total number of the tested ticks within each geographic location and in relation to the examined corresponding tick spp., multiplied by 100. This metric was used to estimate the lower bound of infection at the individual tick level, particularly because samples were analyzed in pools. As prevalence could not be directly determined from pooled testing due to the inability to identify the exact number of infected individuals, MIR was selected as the primary measure of infection frequency in this study.

## 3 Results

### 3.1 Morphological and molecular tick identification

**Figure 2:**
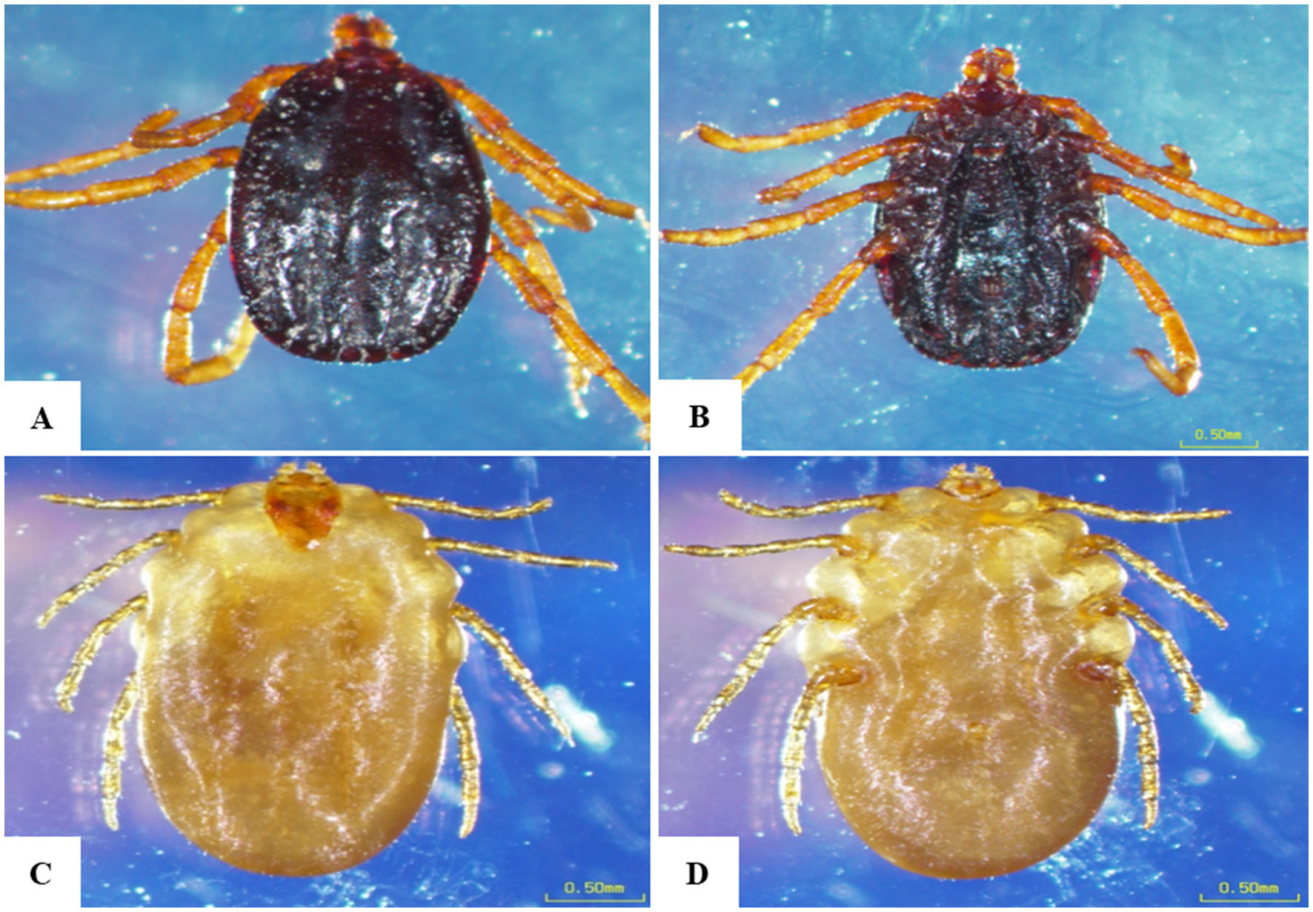
Morphological characteristics of *Rhipicephalus annulatus* obtained in the present study. **(A)** Male, dorsal surface, **(B)** male, ventral surface, **(C)** female, dorsal surface, **(D)** female, ventral surface; scale bar: 0.50 mm.

**Figure 3:**
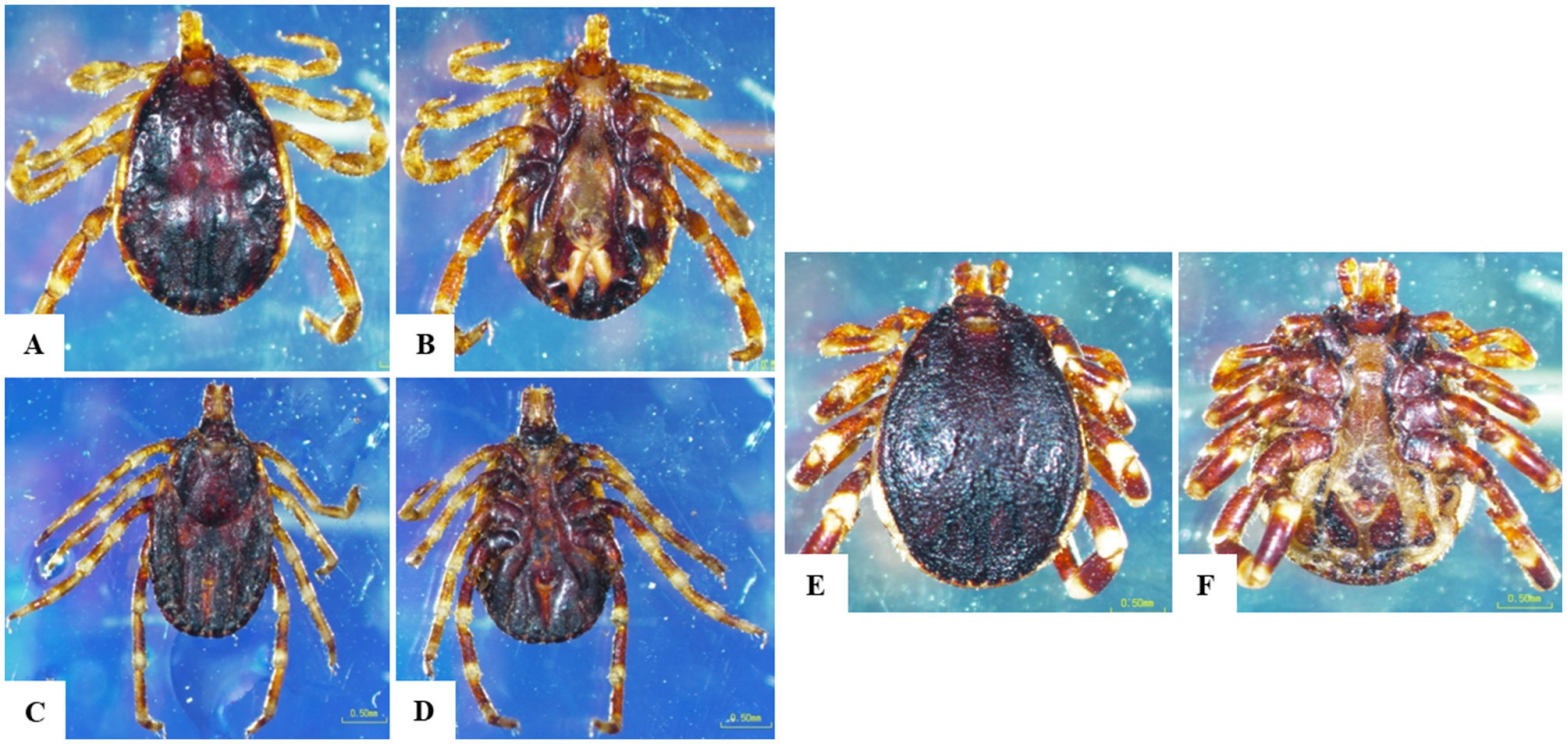
Morphological characteristics of *Hyalomma* species obtained in the current study. **(A)** *H. dromedarii* male, dorsal surface, **(B)** *H. dromedarii* male, ventral surface, **(C)** *H. dromedarii* female, dorsal surface, **(D)** *H. dromedarii* female, ventral surface, **(E)** *H. marginatum* male, dorsal surface, **(F)** *H. marginatum* male, ventral surface; scale bar: 0.50 mm.

We collected 258 ticks from 110 cattle across the study locations. In initial morphological characterization. Specifically, in the Qena governorate, 79 ticks were retrieved from 36 cattle. Broken down by location, we collected 35 ticks from 17 cattle at Deshna (*R. annulatus*: 23 adult females, 11 nymphs; *H. dromedarii*: one adult female), 44 ticks from 19 cattle at Naja' Hammadi (*R. annulatus*: 35 adult females, 8 nymphs; *H. dromedari*: one adult male). At the Girga location in the Sohag governorate, we collected 82 ticks from 34 cattle (*R. annulatus*: 54 adult females, one adult male; *H. dromedarii*: 12 adult males, 15 adult females). At the Esna location in the Luxor governate, we collected 96 ticks from 40 cattle (*R. annulatus*: 68 adult females, 23 nymphs; *H. dromedarii*: one adult male; *H. marginatum*: four adult females). For each species, and at each geographical location, adult females accounted for the largest proportion of collected ticks. Representative examples displaying the morphological characteristics of each collected species are shown in Figures 2, 3.

**Figure 4:**
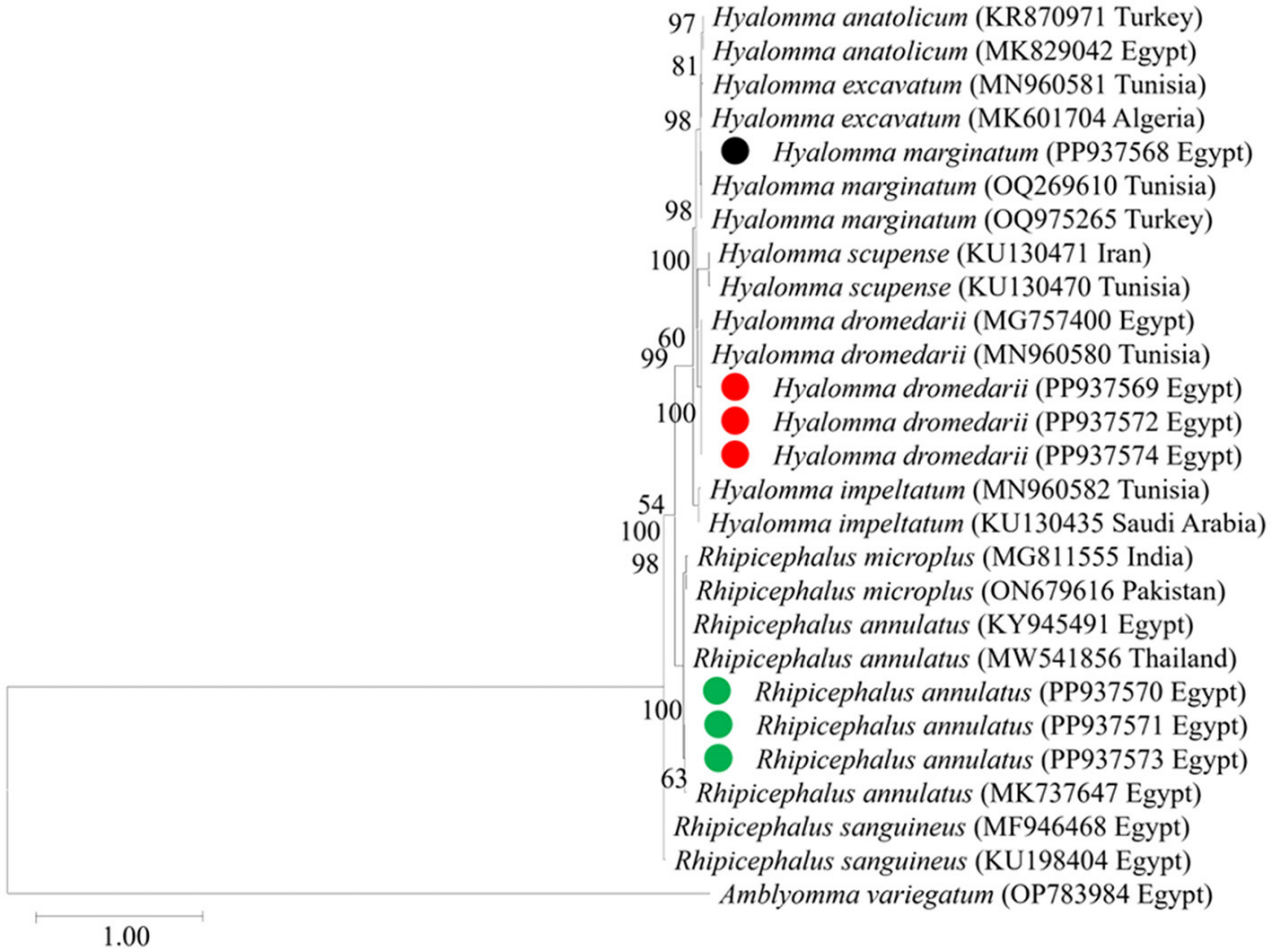
The phylogenetic analysis of tick spp. identified in this study based on *mt-rrs* (16S rRNA) gene sequences. The phylogenetic tree was constructed using the Maximum Likelihood method with a Tamura 3-parameter model, employing MEGA version 11 software. The numbers displayed at the nodes of the tree indicate the percentage occurrence of each clade based on 1,000 bootstrap replications of the data. *Amblyomma variegatum* (OP783984) was used as the out group. The sequences obtained in this study are circled.

We then targeted 177 pooled tick samples for DNA extraction and PCR analysis. Each pooled sample exhibited the expected 455-bp band of the 16S rRNA gene specific to tick species, thus confirming successful DNA extraction. Twenty PCR products representative for each morphologically identified tick species were then subjected to sequencing and phylogenetic analysis as shown in Figure 4. The *H. dromedarii* sequences identified in this study exhibited 99.8% similarity with *H. dromedarii* sequences (accession numbers MG757400 and MN960580) previously obtained from Egypt and Tunisia, respectively. Meanwhile, the *H. marginatum* sequences we obtained displayed a 99.3% similarity with *H. marginatum* sequences from Tunisia and Turkey (accession numbers OQ269610 and OQ975265, respectively). Additionally, the *R. annulatus* sequences we identified exhibited a 99.6% similarity with an *R. annulatus* sequence from Egypt (accession number: KY945491). The obtained sequences for tick species identification were deposited in the GenBank database with accession numbers PP937570, PP937571, and PP937573 for *R. annulatus*, PP937568 for *H. marginatum*, and PP937569, PP937572, and PP937574 for *H. dromedarii*.

### 3.2 Detection of tick-borne protozoa

In this study, 177 pooled tick DNA samples were examined using nested PCR to detect the 18S rRNA gene, which is indicative of piroplasmid species. Seventeen of these DNA samples tested positive, representing a MIR of 9.6%, with each positive finding representing a single bovine host. These 17 positive samples then underwent further PCR testing to determine the MIR of specific piroplasmid pathogens (*B. bovis, B. bigemina*, and *T. annulata*) employing primers specific for each species. A nested PCR assay targeting the *SBP-4* gene of *B. bovis* revealed the presence of this piroplasmid pathogen in 9/17 pooled samples. Contrastingly, *B. bigemina*, was not identified in any sample in an assay targeting its *RAP1a* gene. A conventional PCR assay targeting the *Tams1* gene of *T. annulata* revealed this pathogen was present in 12/17 pooled samples. The MIR for these tick-borne protozoans are displayed together with the absolute numbers of samples in which they were detected, in Table 2.

**Table 2:** Minimum infection rates of the detected protozoa in tick pools by PCR-sequencing assay.

**Location**	**No. of samples**	**Detected protozoa**									
		***Colpodella*** **spp**.		***T. annulata***		***T. orientalis***		***B. bovis***		***B. bigemina***	
		**No**.	**MIR**	**No**.	**MIR**	**No**.	**MIR**	**No**.	**MIR**	**No**.	**MIR**
Qena	79	2	2.5	1	1.3	0	0	2	2.5	0	0
Sohag	82	3	3.7	6	7.3	1	1.2	4	4.9	0	0
Luxor	97	1	1.0	5	5.2	0	0	3	3.1	0	0
Total	258	6	2.3	12	4.7	1	0.4	9	3.5	0	0

No., number of positive tick pool; MIR, minimum infection rate (%).

In *Hyalomma* ticks, MIR was 5.9% for *T. annulata*, 2.9% for *B. bovis*, and 0% (undetected) for *T. orientalis* and *Colpodella* spp. In *R. annulatus* ticks, the MIR was 4.4% for *T. annulata*, 3.3% for *B. bovis*, 0.5% for *T. orientalis*, and 2.7% for *Colpodella* spp. The MIRs for all pathogens in different tick species by sampling location are presented in Table 3. In addition, multiple pathogens were detected within single tick pools. Table 4 provides detailed information on the co-infection rates for the detected protozoa, categorized by the tick collection sites and the specific tick species.

**Table 3:** Minimum infection rates of the detected protozoa in tick pools by PCR-sequencing assay according to tick species in relation to sampling location.

**Protozoa**	**Location (no. of positive pools)**	**Tick species**					
		***H. dromedarii*** ♂	***H. dromedarii*** ♀	***H. marginatum*** ♂	***R. annulatus*** ♂	***R. annulatus*** ♀	***R. annulatus*** **N**
		**No. (MIR)**	**No. (MIR)**	**No. (MIR)**	**No. (MIR)**	**No. (MIR)**	**No. (MIR)**
*Colpodella* spp.	Qena (2)	0	0	0	0	0	2/31 (6.5)
	Sohag (3)	0	0	0	0	3/54 (5.6)	0
	Luxor (1)	0	0	0	0	1/68 (1.5)	0
	Qena (1)	0	0	0	0	1/46 (2.2)	0
*T. annulata*	Sohag (6)	0	2/15 (13.3)	0	0	4/54 (7.4)	0
	Luxor (5)	0	0	0	0	4/68 (5.9)	1/23 (4.4)
	Qena (0)	0	0	0	0	0	0
*T. orientalis*	Sohag (1)	0	0	0	0	1/54 (1.9)	0
	Luxor (0)	0	0	0	0	0	0
	Qena (2)	0	0	0	0	2/46 (4.4)	0
*B. bovis*	Sohag (4)	0	1/15 (6.7)	0	0	3/54 (5.6)	0
	Luxor (3)	0	0	0	0	3/68 (4.4)	0
	Qena (0)	0	0	0	0	0	0
*B. bigemina*	Sohag (0)	0	0	0	0	0	0
	Luxor (0)	0	0	0	0	0	0

No., number of positive tick pool/total number of the corresponding tick species; ♂, male ticks; ♀, female ticks; N, nymphs; MIR, minimum infection rate (%).

**Table 4:** Co-infection rates of the detected protozoa in overall tick pools according to tick collection sites and tick specie.

**Co-detection of different pathogens**	**Qena no. (MIR)**	**Sohag no. (MIR)**	**Luxor no. (MIR)**	***H. dromedarii* no. (MIR)**	***H. marginatum* no. (MIR)**	***R. annulatus* no. (MIR)**
*Colpodella* spp. + *B. bovis*	1/48 (2.1)	0/69	1/60 (1.7)	0/26	0/4	3/146 (2.1)
*Colpodella* spp. + *T. annulata*	0/48	1/69 (1.5)	0/60	0/26	0/4	2/146 (1.4)
*B. bovis* + *T. annulata*	1/48 (2.1)	1/69 (1.5)	1/60 (3.3)	1/26 (3.9)	0/4	1/146 (0.7)
*Colpodella* spp. + *B. bovis*+ *T. annulata*	0/48	1/69 (1.5)	0/60	0/26	0/4	1/146 (0.7)
*B. bovis* + *T. annulata* + *T. orientalis*	0/48	1/69 (1.5)	0/60	0/26	0/4	1/146 (0.7)

No., number of tick pools; MIR, minimum infection rate (%).

### 3.3 Sequencing and phylogenetic analysis

**Figure 5:**
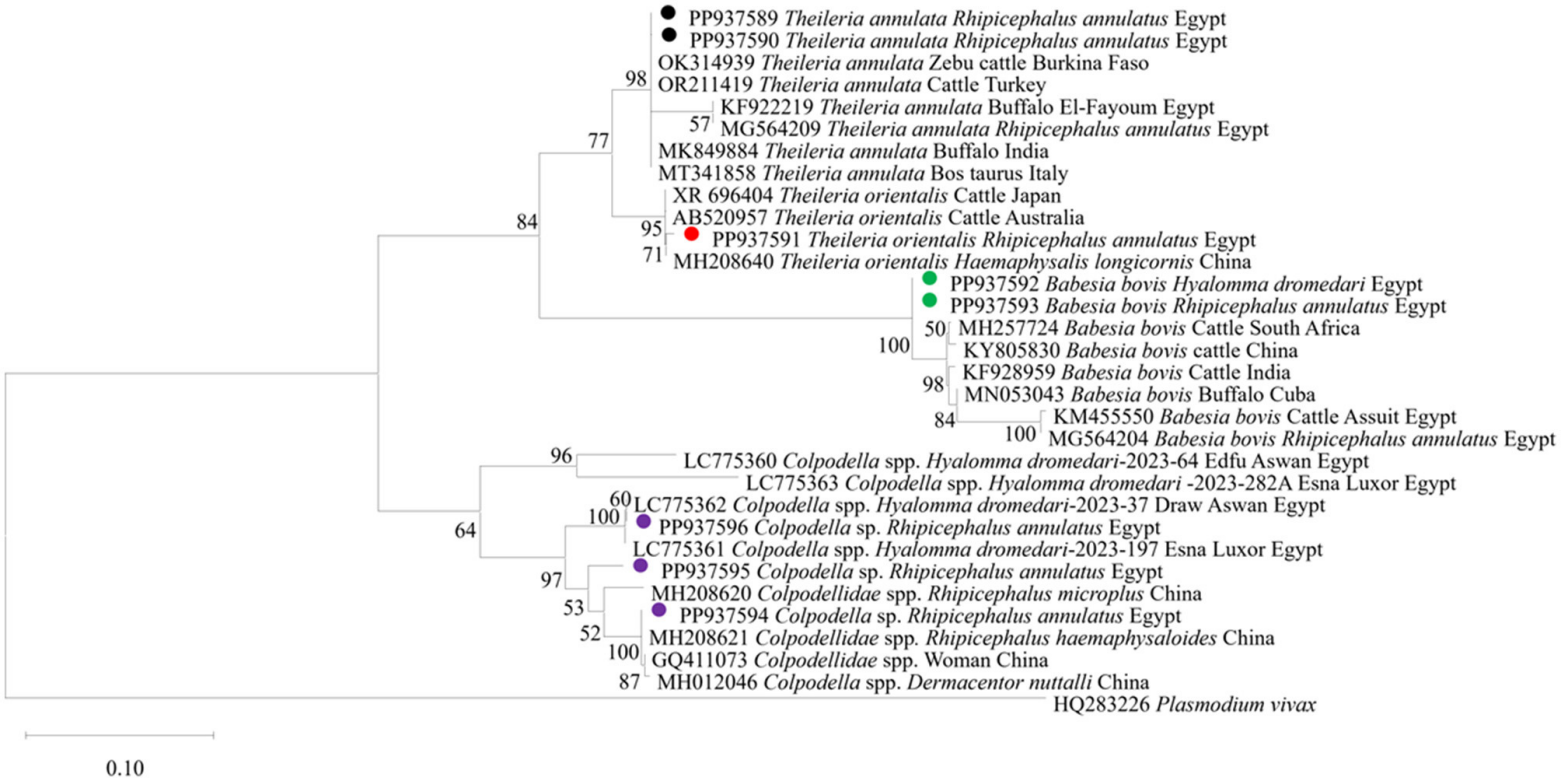
The phylogenetic analysis showing the detected protozoa based on 18S rRNA gene sequences. The phylogenetic tree was constructed using the Maximum Likelihood method with Tamura-Nei model, employing MEGA version 11 software. The numbers displayed at the nodes of the tree indicate the percentage occurrence of each clade based on 1,000 bootstrap replications of the data. *Plasmodium vivax* (HQ283226) was used as out group. The sequences obtained in this study are circled.

**Figure 6:**
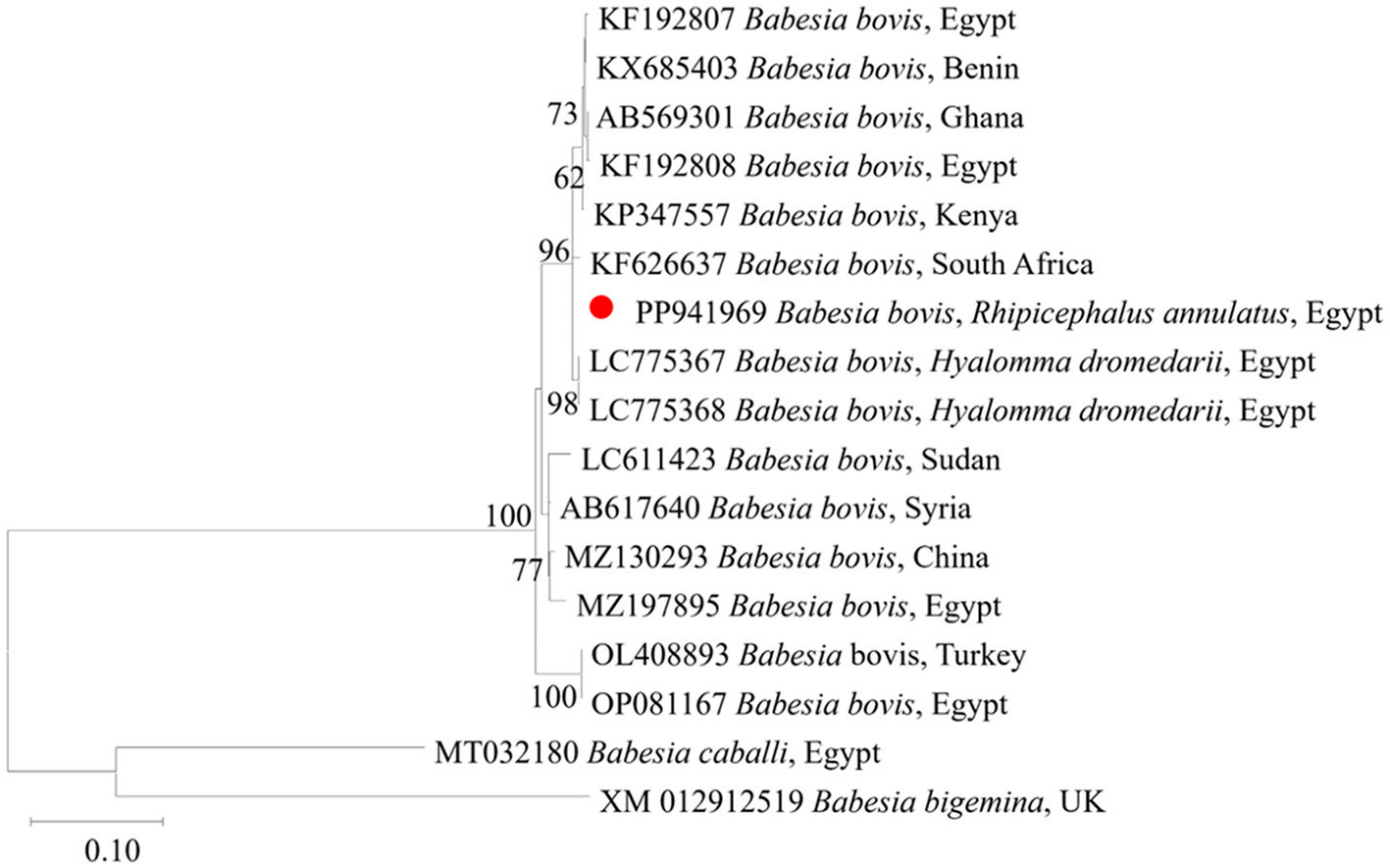
Phylogenetic analysis of *B. bovis* identified in this study based on *SBP4* gene sequences. The phylogenetic tree was constructed using the Maximum Likelihood method with the Tamura 3-parameter model, employing MEGA version 11 software. The numbers displayed at the nodes of the tree indicate the percentage occurrence of each clade based on 1000 bootstrap replications of the data. *Babesia bigemina* (XM012912519) was used as out group. The sequence obtained in this study is circled in red.

**Figure 7:**
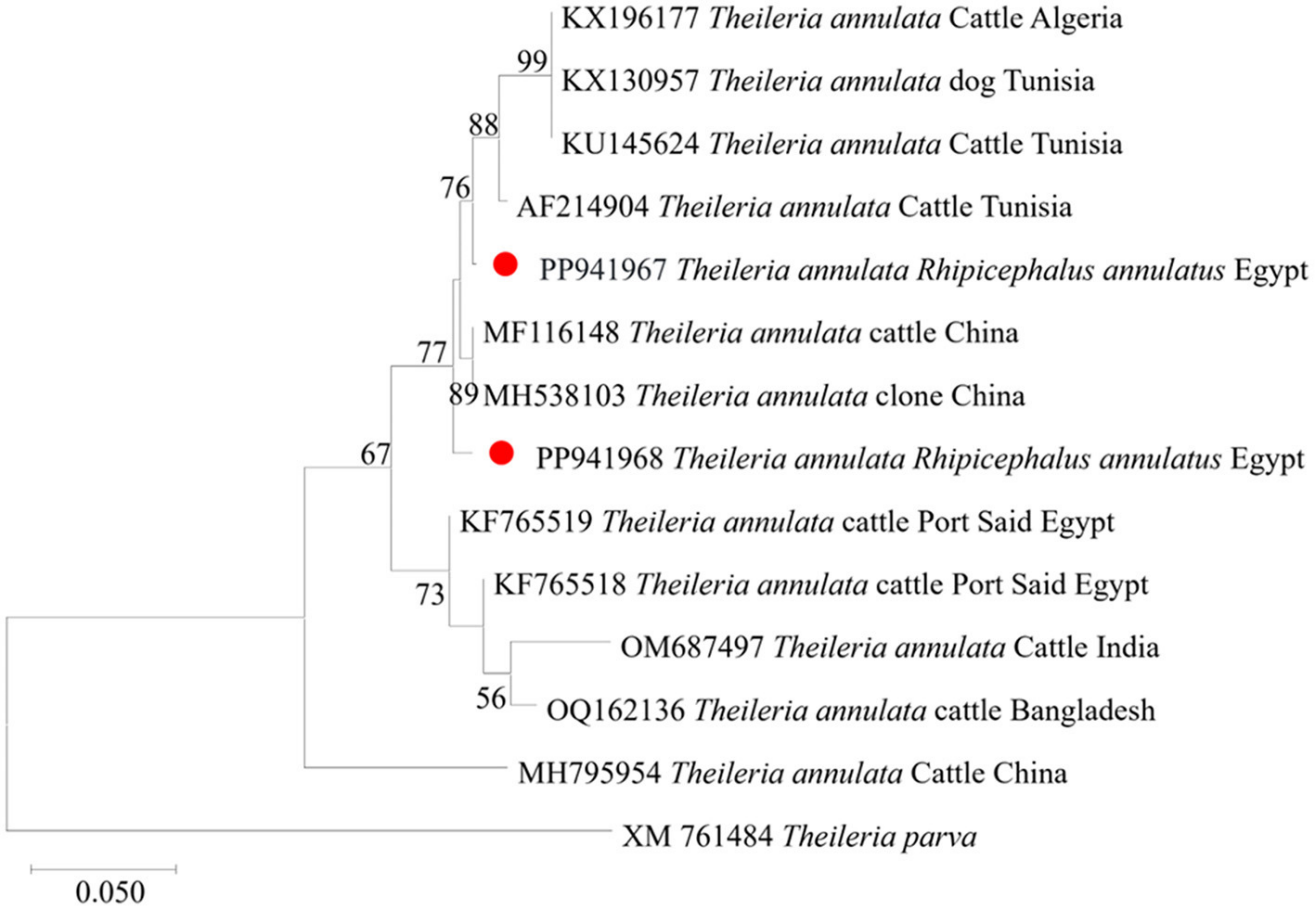
Phylogenetic analysis of *T. annulata* identified in this study based on *Tams 1* gene sequences. The phylogenetic tree was constructed using the Maximum Likelihood method with the Tamura 3-parameter model, employing MEGA version 11 software. The numbers displayed at the nodes of the tree indicate the percentage occurrence of each clade based on 1000 bootstrap replications of the data. *Theileria parva* (XM761484) was used as out group. The sequences obtained in this study are circled in red.

We then submitted the 17 pooled piroplasm (18S rRNA gene)-positive samples described above for direct sequencing. Among the obtained sequences, the numbers exhibiting high similarity with existing sequences in the GenBank database were six for *Colpodella* spp., six for *T. annulata*, four for *B. bovis*, and one for *T. orientalis*. The obtained sequences for the 18S rRNA gene were submitted to the GenBank database with accession numbers PP937594, PP937595 and PP937596 for *Colpodella* spp., PP937591 for *T. orientalis*, PP937589 and PP937590 for *T. annulata*, and PP937592 and PP937593 for *B. bovis*. The phylogenetic tree depicting the evolutionary relationship between the detected protozoa and other apicomplexans is shown in Figure 5. Positive PCR products obtained with *B. bovis* or *T. annulata*-specific primers were selected for sequencing. For *B. bovis* (n = 1), the obtained sequence showed approximately 99% similarity with *B. bovis* sequences previously reported in South Africa (accession number KF626637) and Egypt (accession number LC775376). Furthermore, the obtained sequences for *T. annulata* (n = 2) showed 98.6% identity with *T. annulata* sequences previously reported in Tunisia (accession number AF214904) and China (accession numbers MH538103 and MF116148). We thus confirmed the presence of *B. bovis* and *T. annulata* in the samples analyzed. The obtained sequences were deposited in the GenBank database with accession numbers PP941969 (for *B. bovis SBP-4* gene), or PP941967 or PP941968 (for *T. annulata Tams1* gene). The phylogenetic trees for *B. bovis* and *T. annulata*, which illustrate the relationships and evol.utionary connections between different strains of *B. bovis* and *T. annulata*, are shown in Figures 6, 7, respectively.

## 4 Discussion

Here, we present a comprehensive molecular investigation of ixodid tick infestation in cattle in Upper Egypt, coupled with a detailed molecular characterization of the hemoprotozoan parasites identified in ticks infesting cattle in this region. To the best of our knowledge, this study provides the most recent epidemiological data on ticks and associated tick-borne pathogens in the cattle population in southern Egypt, including crucial data on protozoan parasites (specifically, *Colpodella* spp.) that have only recently been in identified Egypt, in ticks infesting another livestock species (camels).

All collected ticks were classified as *H. dromedarii, H. marginatum*, or *R. annulatus* based on morphological criteria (Figure 3). Because morphological identification can be challenging when dealing with engorged ticks (21, 30), we employed a molecular technique (31) targeting the protozoan 16S rRNA gene, to validate tick species identification (2, 31–34). In our phylogenetic analyses, sequences for morphologically identified tick species formed distinct clusters, validating our identification of *H. dromedarii, H. marginatum*, and *R. annulatus* (Figure 4).

The current study provides further molecular evidence of *Colpodella* spp. within ixodid ticks in Upper Egypt, following our recent identification of this parasites in *H. dromedarii* ticks infesting camel, in the first such report in Egypt (11). Here, we identified *Colpodella* spp. in ticks infesting cattle, specifically *R. annulatus*, at an MIR of 2.7%. We successfully amplified 1,550-bp DNA fragments of the 18S rRNA gene associated with *Colpodella* spp., showing sequence identities between 95% and 100% with *Colpodella* spp. entries in the GenBank database (Figure 4). Our phylogenetic analysis placed the *Colpodella* spp. sequences in a sister group to the apicomplexan clade, encompassing microorganisms such as *Babesia* spp. and *Theileria* spp. The phylogenetic tree (Figure 5) demonstrated that the *Colpodella* spp. sequences obtained from *H. dromedarii* ticks previously recorded in Aswan and Luxor governorates of Egypt (LC775360, LC775361, LC775362, and LC775363) clustered tightly together, forming a well-supported clade with high bootstrap values (96%−100%), indicating a close genetic relationship among these isolates. This monophyletic grouping may reflect a conserved lineage of *Colpodella* spp. specifically associated with *H. dromedarii* in southern Egypt. In contrast, the sequences PP937595 and PP937596 derived from *Rhipicephalus annulatus* ticks, in this study in different locations in southern Egypt, showed a separate clustering pattern, positioned closer to *Colpodella* sequences from Chinese *Rhipicephalus* species (e.g., MH208620, MH208621), suggesting potential host- or region-specific genetic divergence. These results imply that *Colpodella* spp. may exhibit host-specific genotypes and that their genetic diversity is influenced by tick species and possibly ecological factors. The phylogenetic proximity of Egyptian and Asian isolates further raises the possibility of a broader transboundary distribution or ancient lineage conservation, warranting further investigation into the evolutionary dynamics and zoonotic potential of these protozoans.

Our findings provide further evidence on the presence of *Colpodella* spp. in ixodid ticks across Upper Egypt, although this presence does not necessarily indicate biological transmission from ticks to cattle. The cattle infested with *Colpodella*-carrying ticks in this study were all clinically healthy at the time of tick collection. Our findings suggest that cattle, like camels, could serve as a valuable sentinel species for detecting emerging tick-borne diseases, such as *Colpodella* spp. infections. A critical concern related to *Colpodella* spp. is its zoonotic potential, as documented in reports from China (15, 17). Further studies must address the transmission mechanism, pathogenicity, and zoonotic potential of this emerging pathogen. Importantly, research is needed to develop protective measures for individuals who are in frequent proximity to animals susceptible to tick infestations. Protozoan parasites in the genus *Colpodella* represent an emerging health threat that appears to have a wider geographical reach than previously thought, and they may induce a disease with severe symptoms in humans (based on the small number of cases reported so far).

The MIR for piroplasma microorganisms we detected in pooled cattle-infested ticks (one pool collected from one bovine host) in this study was 9.6%, which is lower than the rates reported in tick-infested cattle populations in other regions of Egypt. Specifically, previous studies have reported rates of 52.5% in cattle in El-Wady El-Gadid governorate (35), 17% in household cattle across Beni-Suef, Qalyubia, El-Wady El-Gadid, Qena, and Behera governorates (36), and 20% in cattle in Qena and Sohag governorates (37).

Among the individual piroplasma species, we found *B. bovis* at an MIR of around 3.5% in our pooled tick samples, specifically 3.3% in both *H. dromedarii* and *R. annulatus* ticks. As with the findings for piroplasma microorganisms overall, higher infection rates for these pathogens have been reported in cattle; specifically, 55% in Giza governorate (38), 4% in Beheira and Faiyum governorates (5), 52.4% in Assiut governorate (39), 9% in Qena and Sohag governorates (40), 3.2% in Behera and Menofia governorates (41), and 5.3% in Qena and Sohag governorates (37). We did not detect *B. bigemina* in any sample in our study, although this pathogen has previously been reported in Egyptian cattle populations at infection rates ranging from 1.1% to 66% (5, 36, 37, 40, 42, 43).

In the present study, *T. annulata* was detected in our samples with an MIR of 4.7%; specifically, 6.7% and 4.5% in *H. dromedarii* and *R. annulatus* ticks, respectively. Our results are consistent with findings in previous studies Dahl on this pathogen in Egypt. Notably, previous studies have recorded infection rates in cattle of 9.6% in Menoufia, Behera, Giza, and Sohag governates (42), 63.6% in El-Wady El-Gadid governate (19), 22% in Beni-Suef, Faiyum, and El-Wady El-Gadid governates (43), 15.9% in Beni-Suef, Qalyubia, El-Wady El-Gadid, Qena, and Behera governorates (36), 18.1% in Faiyum, Assiut, and Kharja governates (9), 9.6% in Behera and Menofia governorates (41), and 10.7% in Qena and Sohag governorates (37). We detected *T. orientalis* in one pooled *R. annulatus* tick sample collected from one bovine host in the Sohag governorate (representing an MIR of 0.5%). This finding aligns with previous research, including the reports by (41), who found a rate of 0.68% in the Behera and Menofia governorates, and (44), who found an infection rate of 8.8% in northern Egypt. The presence of *T. orientalis* in *R. annulatus* ticks suggests a potential route of infection for this piroplasmid microorganism. We speculate that there are regional variations in the spread of this pathogen across Egypt, but this requires further investigation. Multiple pathogens were identified in both pooled tick samples and individual ticks. However, it is crucial to note that detecting various pathogens in pooled samples does not necessarily indicate that individual ticks were co-infected; such findings could result from several mono-infected ticks being present in a single pool. Our results highlight the benefits of molecular techniques, which offer the advantage of detecting a wide range of pathogens in ticks (6, 20).

This study has a number of limitations. As a cross-sectional study, its design did not allow for investigation of seasonal fluctuations in tick population densities, the impact of animal movement on tick dispersal, or the associated challenges in correlating infection and infestation risk factors. Since the study was conducted in a specific region, Upper Egypt, we cannot draw any conclusions on the tick-related health threats in other regions across the country. To comprehensively evaluate the current health risk posed by ticks and tick-borne pathogens in Egypt, longitudinal research spanning a wider geographic area and invol.ving a greater number of samples, including samples collected from ticks infesting humans, is imperative. Furthermore, there is a lack of specific primers for detecting *Colpodella* spp. using genes other than the 18S rRNA, and this represents a significant limitation for this research. Addressing this constraint requires future studies to integrate whole genome sequencing of *Colpodella* spp., facilitating the development of appropriate specific primers.

## 5 Conclusion

In conclusion, we elucidated pathogenic piroplasms carried by ixodid ticks infesting cattle in southern Egypt. Specifically, we detected *Colpodella* spp. in *R. annulatus* ticks retrieved from infested cattle, following our previous detection of this pathogen in *H. dromedarii* ticks infesting camel in a similar study area. *T. orientalis* was detected in *R. annulatus* ticks, suggesting this tick species may act as a vector for transmitting the pathogen to cattle in Egypt. Although we identified piroplasms in ticks, we cannot confirm their biological transmission to cattle in this study, and further surveillance and control measures to mitigate the risk of tick-borne diseases in animal and human populations are crucial. Here, we provide important evidence on the MIR of ticks and tick-pathogens that may affect cattle in Upper Egypt.

## Data Availability

All data supporting the findings of this study are presented in the article. The sequences obtained for tick species identification have been deposited in the GenBank database under accession numbers PP937568-PP937574. The 18S rRNA gene sequences are also available in GenBank, with accession numbers PP937594, PP937595, and PP937596 for *Colpodella* spp., PP937591 for *T. orientalis*, PP937589 and PP937590 for *T. annulata*, and PP937592 and PP937593 for *B. bovis*. Additionally, the *SBP-4* gene for *B. bovis* is deposited under PP941969, while the *Tams1* gene for *T. annulata* is listed under PP941967 and PP941968.
